# Thionated organic compounds as emerging heavy-atom-free photodynamic therapy agents

**DOI:** 10.1039/d0sc04747c

**Published:** 2020-09-22

**Authors:** Luis A. Ortiz-Rodríguez, Carlos E. Crespo-Hernández

**Affiliations:** Department of Chemistry, Case Western Reserve University Cleveland Ohio 44106 USA carlos.crespo@case.edu

## Abstract

This minireview focuses on recent progress in developing heavy-atom-free photosensitizers based on the thionation of nucleic acid derivatives and other biocompatible organic compounds for prospective applications in photodynamic therapy. Particular attention is given to the use of thionated nucleobase derivatives as “*one-two punch*” photodynamic agents. These versatile photosensitizers can act as “*Trojan horses*” upon metabolization into DNA and exposure to activating light. Their incorporation into cellular DNA increases their selectivity and photodynamic efficacy against highly proliferating skin cancer tumor cells, while simultaneously enabling the use of low irradiation doses both in the presence and in the absence of molecular oxygen. Also reviewed are their primary photochemical reactions, modes of action, and photosensitization mechanisms. New developments of emerging thionated organic photosensitizers absorbing visible and near-infrared radiation are highlighted. Future research directions, as well as, other prospective applications of heavy-atom-free, thionated photosensitizers are discussed.

## Abstract 2

This minireview focuses on recent progress in developing heavy-atom-free photosensitizers based on the thionation of nucleic acid derivatives and other biocompatible organic compounds for prospective applications in photodynamic therapy.

## Graphical abstract

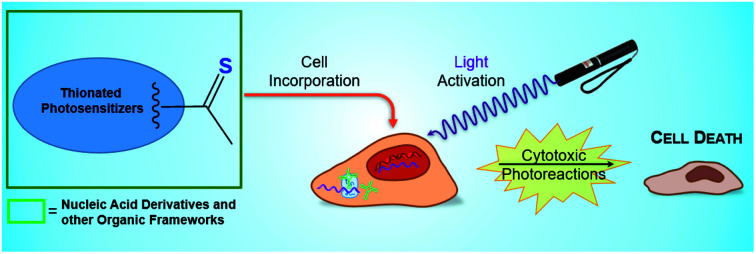

## 1. Introduction

### 1.1. Skin cancer and photodynamic therapy

Skin cancer is pervasive around the world.^1–3^ In the USA, it is estimated that more than 3 million Americans are affected with non-melanoma skin cancers (NMSC) annually, including basal cell carcinoma (BCC) and squamous cell carcinoma (SCC).^4,5^ Between 1976–1984 and 2000–2010, the overall incidence of BCC increased by 145%, while it increased by 263% for SCC over the same period.^6^ Likewise, actinic keratosis (AK) is a common precancerous skin condition caused by DNA damage in the keratinocytes of the epidermis due to chronic exposure to sunlight. AK is of major public health concern because of its high prevalence, substantial financial impact, and potential for malignant transformation. In 2004, AK prevalence was estimated at almost 40 million in the USA, with an estimated annual cost of $1.2 billion in healthcare.^7,8^ AK can develop into keratinocyte carcinoma (KC), including BCC and SCC of the skin, which are the most common cancers in the USA and in other countries with predominantly light-skinned populations. Of particular concerns are organ transplant patients who have a higher risk of AK and KC lesions than the general population due to their chronic immunosuppressed state.^9^ AK lesions are associated with the development of SCC, which are more aggressive, proliferate faster, and metastasize more often in organ transplant patients. It is estimated that the risk of SCC increases by up to 65–250 times in organ transplant patients compared to immunocompetent patients.^9–11^

Solar radiation is the major environmental factor in the development of BCC and SCC.^12^ DNA damage is directly implicated in the initiation of these cancers. In particular, the DNA cyclobutane pyrimidine dimers and (6-4) pyrimidine–pyrimidone photoproducts that are generated when DNA absorbs ultraviolet radiation are associated with mutagenesis and cancer. Individuals with xeroderma pigmentosum, a condition in which the nucleotide excision repair pathway is disabled by mutation, are unable to remove mutagenic DNA photoproducts from their skin cells and have a *ca.* 2000-fold increased rate of skin cancer in sun-exposed areas.^13^

Photodynamic therapy (PDT) has key advantages over other traditional therapies such as surgery, chemotherapy, and radiotherapy for the treatment of NMSC and AK and KC lesions.^14–17^ As a treatment that primarily uses a photoactivable drug (a.k.a., photosensitizer) to generate singlet oxygen and other reactive oxygen species (ROS), it offers a high degree of spatiotemporal selectivity in tumor destruction, noninvasiveness, and reduced side effects. Furthermore, it is easy to combine with other therapies. Photosensitizers are often classified as porphyrin-derived and non-porphyrin photosensitizers. Porphyrin-derived photosensitizers are classified as first, second, and third generation photosensitizers. The first generation includes hematoporphyrin derivatives and the clinically approved Photofrin®. The second generation corresponds to those porphyrin-derived photosensitizers that absorb at longer wavelengths and exhibit less skin photosensitization than their predecessors.^18^ When second generation photosensitizers are bound to carriers, such as antibodies to increase their accumulation into malignant tissue, they are classified as third generation photosensitizers.^19^ The study and development of non-porphyrin derivatives have been significantly slower. Non-porphyrin derivatives that have been investigated recently include cationic photosensitizers, such as methylene blue, toluidine blue and other chalcogenopyrilium dyes, phenothiazinium, and benzo[*a*]phenothiazinium derivatives.^20^

PDT has grown in popularity in dermatology, mainly due to the easy accessibility of the skin to light exposure and the simplicity of topical use of photosensitizers. In PDT, neither the drug nor the photoactivating wavelength should have the desired therapeutic effect on its own. Once the drug accumulates in the target tissue, the photodynamic process is initiated by the localized application of radiation at wavelengths that correspond to the absorption spectrum of the photosensitizer (*e.g.*, with optical fibers). Topical PDT offers a superior cosmetic outcome than conventional therapies such as surgery, radiation therapy, and chemotherapy, which can cause serious side effects by the loss of normal cell function due to nonspecific targeting of the treatments.^1,21^ PDT is approved for the treatment of wide-ranging NMSC in Europe, USA, and in other countries,^21–23^ and has demonstrated high efficacy for AK, superficial and nodular BCC, squamous cell carcinoma *in situ* or Bowen's disease, and field cancerization.^22^ It has also been recommended for use in photo-rejuvenation, acne treatment, and other skin conditions.^23^

PDT also has disadvantages. The disadvantages include severe pain and adverse reactions,^24,25^ such as persistent photosensitization.^26,27^ In general, the efficacy of PDT depends on the type of target cells and their oxygenation status, the photosensitizer used and its ability to penetrate the targeted diseased tissue selectively, the light source and the wavelength of light activation required, and the duration of the light-irradiation treatment. The efficacy of most clinical photosensitizers such as those discussed above strictly depends on the concentration of O_2_ available. The PDT process itself also consumes O_2_, thereby effecting tumor hypoxia, which can increase the tumor's resistance to PDT.^28,29^ The O_2_ concentrations in solid tumors also vary widely by location due to the aggressive proliferation of cancer cells and insufficient blood supply, with some interior regions of the tumor exhibiting O_2_ concentrations of less than 4% and could even decrease to 0% locally, which severely limits the efficacy of PDT against *in vivo* hypoxic tumors.^30,31^ Furthermore, the photosensitizers often incorporate transition metals in their structures to increase their triplet yields. Besides poor selectivity for the target tissue, such metal complexes frequently suffer from high cytotoxicity in the absence of light activation and are often costly and difficult to synthesize.^32–35^ In view of these drawbacks, there is a clinical need to develop highly selective, heavy-atom-free photodynamic agents for PDT.

In general, to minimize the side effects, the photosensitizer should have excellent biocompatibility and photostability with no dark toxicity, large absorption coefficients in the optical window to be used for PDT, and should exhibit appropriate retention time in living tissues with relatively rapid clearance from the body.^36,37^ In addition, the photosensitizer should be widely available with a high degree of chemical purity and good stability to allow for prolonged storage, and should be inexpensive and simple to synthetized.^36^

### 1.2. Photochemical mechanisms and PDT-mediated cytotoxicity

**Fig. 1:**
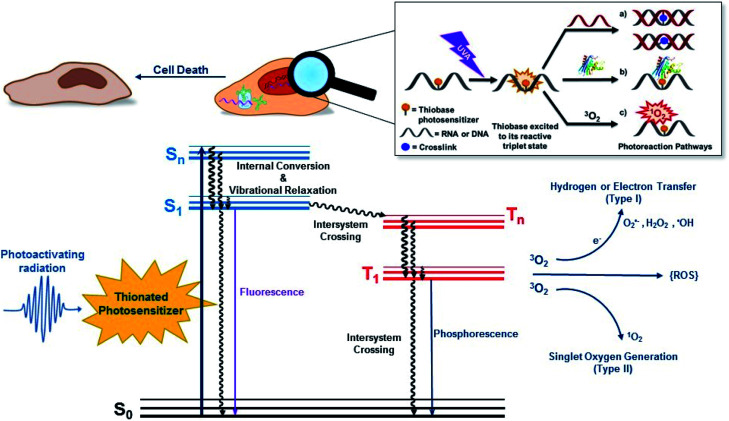
(Top) Generic UVA-induced reaction mechanisms of the thiobase derivatives, which includes photocrosslinking reactions with (a) DNA or (b) proteins, and (c) triplet-energy transfer to generate molecular oxygen in its excited singlet state. Electron transfer from the triplet state of the thiobase to molecular oxygen to generate the superoxide anions and other ROS such as hydrogen peroxide and hydroxyl radicals is also possible, but not shown. These reactions can lead to cell death. (Bottom) Jabłoński electronic-energy diagram depicting the primary events leading to type I and type II photosensitized reactions, which eventually can result in oxidatively-induced cell damage. S_0_ represents the ground electronic state of the photosensitizer, S_*n*_ and T_*n*_ represent upper excited singlet and triplet states, respectively, and S_1_ and T_1_ are the lowest-energy singlet and triplet states.

Conventional PDT requires the presence and interaction of three key elements: a photosensitizer, light, and O_2_. As depicted in Fig. 1, the photosensitizer is excited to an excited singlet state (S_*n*_) upon exposure to specific wavelengths of light, which can decay radiatively or nonradiatively back to the ground state through internal conversion or can intersystem cross to the triplet manifold. The population reaching the triplet manifold usually internally converts to the lowest-energy and longer-lived triplet state (T_1_). Once in the T_1_ state, the photosensitizer can react directly or indirectly with surrounding biomolecules. In a type I photosensitization mechanism, hydrogen abstraction or electron transfer between the T_1_ state of the sensitizer and nearby biomolecule occurs, forming free radicals. The radicals may react with molecular oxygen to produce ROS such as hydrogen peroxide (H_2_O_2_), hydroxyl radical (OH˙) and superoxide anions (O_2_˙^−^). In the type II mechanism, the energy of the T_1_ state is transferred to O_2_ to generate singlet oxygen (^1^O_2_) and other ROS such as superoxide ions.^38,39^ Dismutation or one-electron reduction of O_2_˙^−^ gives hydrogen peroxide (H_2_O_2_), which in turn can undergo another one-electron reduction to form highly-oxidant hydroxyl radical species (HO˙). ROS can react with intracellular lipids, proteins, and DNA and RNA molecules to cause cell death. However, their short lifetimes (≤3.5 μs) and limited diffusion distance (≤2 μm), severely restrict their phototoxicity to the intracellular location of the photosensitizer.^36,40,41^ When the photosensitizer is incorporated in DNA or RNA, its T_1_ state can also react with an adjacent nucleobase through [2 + 2] photocycloaddition reactions and/or participate in other photocrosslinking reactions.

PDT-mediated cytotoxicity occurs *via* the three main morphologies: apoptotic, necrotic and autophagy-associated cell death.^36,42^ The subcellular localization of the photosensitizer in different organelles (*e.g.*, nucleus, mitochondria, lysosomes, endoplasmic reticulum, plasma membrane, *etc.*) plays a major role in the cell death mechanism that dominates, but other factors such as the overall PDT dose and the possible metabolization of the photosensitizer into cellular DNA are also important. In general, apoptosis is the principal modality of cell death when cells are treated with PDT *in vitro*.^36^

## 2. Thiobases as heavy-atom-free photosensitizers: “*one-two punch*” photosensitizers to increased tumor selectivity and cellular DNA damage

**Fig. 2:**
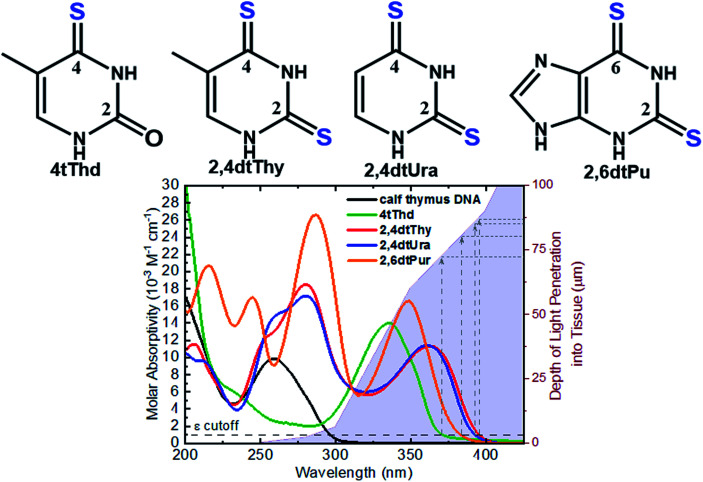
Molecular structure of the thiobases and the molar absorptivity spectra of calf thymus DNA, 4tThd, 2,4dtThy, 2,4dtUra and 2,6dtPu in PBS at pH 7.4 overlaid with depth of light penetration into fair Caucasian skin.^51–53^

The aim of PDT is to kill the tumor cells with minimal effects on healthy surrounding tissue. As discussed above, existing approaches have drawbacks, and there is a clinical need to develop alternatives offering improved target cell selectivity and the ability to work through more than one mechanism besides ROS generation. Site-selected sulfur-substituted nucleobases (a.k.a., thiobases) are a prospective class of heavy-atom-free organic biomolecules for clinical and cosmetic phototherapy applications,^43–48^ in which a sulfur atom replaces the oxygen atom of an exocyclic carbonyl group (Fig. 2). The unique structural, biochemical, and photochemical properties of thiobases offer an attractive strategy for developing highly effective and highly targeted phototherapeutic compounds, working both in the absence and in presence of O_2_.^45,47,49^ A single-atom-substitution converts most of the nucleobases into effective UVA chromophores (*ε* ≥ 10^4^ M^−1^ cm^−1^) that exhibit red-shifted absorption maxima from *ca.* 320 to 380 nm and with absorption bands extending all the way to the near visible region (see, for instance, Fig. 2).^49,50^ One of most attractive applications of thiobases is for topical PDT of hyperproliferative skin conditions that are readily accessible to UVA irradiation. While thiobases have been proposed as prospective UVA photosensitizers for treatment of skin malignancies,^43–47^ they are yet to be used in clinical settings.

4-Thiothymine (4tThd) is the thiobase-photosensitizer that has been studied the most and in a wider-range of cell lines and tissues. 4tThd is metabolized into DNA *via* the thymidine kinase-mediated pyrimidine nucleoside salvage pathway,^48^ and can effectively damage cellular DNA following exposure to nontoxic doses of UVA radiation, leading to cell death by apoptosis.^54^ Thymidine kinase is up-regulated during S-phase^48^ and is more active in rapidly dividing cells.^55,56^ This property can be effectively and conveniently used for therapeutic applications, because the metabolization of nucleobase derivatives into DNA *via* pyrimidine or purine nucleoside salvage pathways is a broadly used therapeutic strategy.^43^ As this process is strictly dependent on phosphorylation of the nucleoside derivative by thymidine kinase, it is particularly highly active in rapidly dividing cancer cells, which exhibit very high proliferation rates compared to normal cells. Indeed, 4tThd and other thiobase derivatives has been demonstrated to be effective in treating hyperproliferative human epidermoid carcinoma cells such as SCC.^12,45–47,57,58^ While 4tThd is not detectably toxic by itself in cultured human cells,^48^ human cell lines treated with 4tThd are killed by UVA radiation doses that are well below the doses required to cause direct cell-death or mutations.^48^ UVA sensitization factors of ∼100-fold can be achieved,^12,48^ which seems to be largely independent of the p53 tumor suppressor status.^12^ Selective sensitization of rapidly dividing cells and p53 independence are both crucial properties for a treatment aimed at cancers in which p53 is often mutated or absent. The p53 tumor suppressor is a sequence-specific DNA-binding protein that is frequently mutated in human cancers and controls the expression of many genes in response to diverse stress stimuli such as UV radiation.^59,60^ Low cytotoxicity of 4tThd has been confirmed in numerous human cell lines, including human fibroblasts and keratinocytes, and SCC, as well as, in rat bladder carcinoma cells.^12,48,61^ 4tThd has also been shown to be a poor mutagen^43,56^ and can be easily introduced into the DNA of cultured cells simply by adding it into the growth medium.

Recent *in vitro* studies with doubly-substituted thiobases such as 2,4-dithiothymidine (2,4dtThy) and 2,6-dithiopurine (2,6dtPu) have shown superior photosensitization efficacy than 4tThd to treat human epidermoid carcinoma cells in combination with nonlethal doses of UVA radiation.^45–47^ Importantly, the efficacy of 2,6dtPu in decreasing the proliferation of SCC is nearly twofold greater than that of 2,4dtThy under equal experimental conditions.^47^ These investigations have demonstrated that doubling thionation of the nucleobases shifts the absorption spectra to longer UVA wavelengths of light and increases the intersystem crossing rate to populate the reactive triplet state compare to 4tThd and other singly sulfur-substituted derivatives. Shifting the absorption spectrum to longer wavelengths is an important requirement for the use of thiobases in phototherapeutic applications because longer wavelengths penetrate correspondingly deeper into tissues (see, Fig. 2), thus allowing skin cancer cells to be treated by photoactivating thiobases at much greater depths within the dermis and epidermis of the skin.^51–53^ Further investigations are require to understand whether these dithionate nucleobases are metabolized into cellular DNA and their specific modes of action.

### 2.1. Photosensitization mechanism of thiobases

**Fig. 3:**
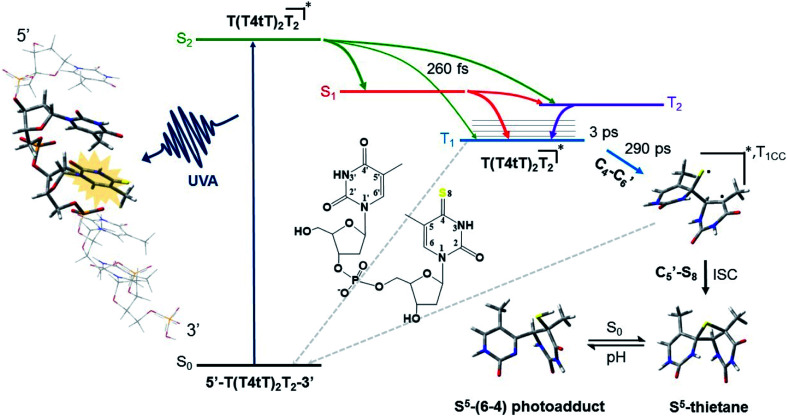
Proposed mechanism for the formation of the DNA (6-4) photoadduct in PBS at pH 7.4. A 4-thiothymine (4tT) base is highlighted with light orange in the single-stranded DNA sequence depicted to the left to imply that 4tT bases are selectively photoactivated by the UVA radiation. 4tThd and the thymidine 5′ relative to the 4tT are represented as tube in the single-stranded DNA sequence, while the other nucleobases are depicted as wireframe in order to highlight the importance of having a thymine 5′ relative to the 4tT for the reaction to occur. Figure reproduced from ref. 63.

**Fig. 4:**
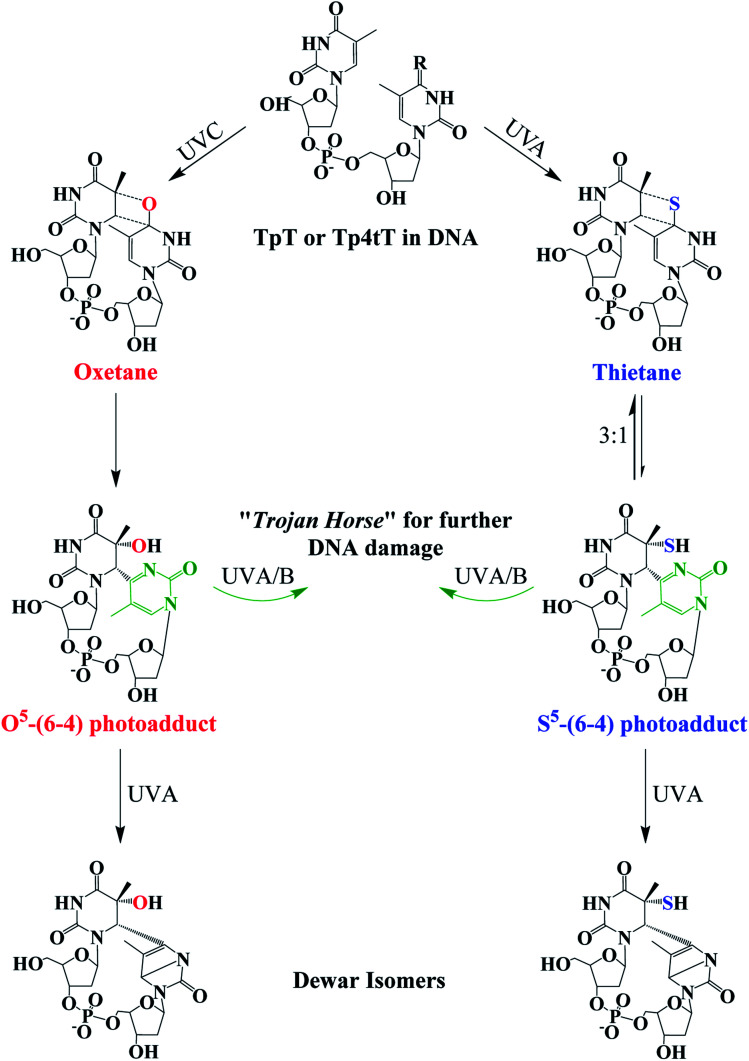
Photochemistry of adjacent thymine–thymine (TpT) or thymine-4-thiothimine (Tp4tT) in DNA upon UV irradiation (modified from ref. 43, 63 and 66). Irradiation of TpT with UVC, or Tp4tT with UVA radiation, generates an oxetane or a thietane intermediate, respectively. The oxetane is highly unstable and spontaneously converts into the O^5^-(6-4) photoadduct (its ring-open form), whereas the thietane is more stable and exists as a 3 : 1 equilibrium between the thietane and its ring-open form; the S^5^-(6-4) photoadduct analog. Irradiation of the ring-open forms of both O^5^-(6-4) photoadduct and S^5^-(6-4) photoadduct with UVA/B radiation can convert them into their respective Dewar isomers or can excite the pyrimidone moiety (highlight with green color) to generate further damage to DNA. Note that metabolization of 4tThd into DNA can be thought as a “Trojan horse” to photoactivate cellular damage (see, Fig. 1), while the formation of the S^5^-(6-4) photoadduct in cellular DNA can further generate DNA damage upon UVA/B excitation of its pyrimidone moiety; a “*one-two punch*” photosensitizer for increased selectivity and efficacy in PDT.

The mechanism by which thiobases photosensitizes induce cellular damage has not been fully elucidated,^43^ but 4tThd has been studied in more detail than any other thiobase currently under consideration for PDT. Importantly, photoactivation of 4tThd, as well as other thiobases,^49^ with UVA radiation can damage DNA through multiple mechanisms (Fig. 1).^45,62–64^ In solution 4tThd generates ^1^O_2_ in high yields,^45,64^ while its photoreactivity in DNA oligonucleotides and cellular DNA is predominantly consistent with an O_2_-independent mechanism.^56,63,65^ The photoreactivity of 4tThd in DNA also depends sensitively on the sequence context. It has been shown to be more photoreactive when it is placed at the 3′ position of thymine, forming a DNA intrastrand crosslink with the thymine placed at the 5′ position.^54,63^ Recent investigations have demonstrated that when 4tThd is incorporated in a single-stranded DNA oligonucleotide, selective photoactivation of 4tThd results in an efficient, sub-1 ps intersystem crossing to populate its reactive triplet state. A [2 + 2] photocycloaddition reaction from the triplet state of 4tThd with an adjacent thymine base in the DNA oligonucleotide leads to the population of a triplet minimum of the thietane intermediate in as short as 3 ps (Fig. 3), which intersystem crosses to its ground state and rearranges to form the (6-4) photoadduct.^63^ Cells defective in repairing S^5^-(6-4) pyrimidine–pyrimidone DNA photoadducts are particularly sensitive to photodynamic treatment with 4tThd,^56^ which indicates that this S^5^-(6-4) photoadduct lesions resembling the canonical (6-4) pyrimidine-pyrimidinone photoadducts contribute significantly to the cytotoxicity. The pyrimidone moiety in the S^5^-(6-4) photoadduct may also act as a *Trojan horse*,^63,66,67^ potentially leading to the formation of secondary cyclobutane pyrimidine dimer or oxidatively-generated damage of neighbor bases in 4tThd-containing single- and double-stranded DNA.^63^ Collectively, the available evidence suggests that UVA irradiation of metabolized 4tThd produces a thietane intermediate in DNA^56,63,68^ upon photoreaction with a consecutive 3′-thymine base that contributes to cell death.^43,48,56^ The formation of the S^5^-(6-4) photoadduct in DNA upon photoactivation, together with the possibility for the pyrimidinone moiety of this lesion to further absorb another UVA or UVB photon to generate further cellular DNA damage (Fig. 4), is the rationale behind naming them as “*one-two punch*” photosensitizers for selective tumor cells death.

The thietane intermediate has been shown to be in a 3 : 1 equilibrium with a S^5^-(6-4) photoadduct lesion (Fig. 4),^62,69^ whereas the oxetane homolog is thought to be unstable and readily converts to its ring open form.^43^ The ring open forms of both the thietane and the oxetane can further convert into their Dewar valence isomer following subsequent exposure to UVA radiation. While the canonical Dewar isomers have been implicated in solar mutagenesis by UVA exposure,^70^ the fate of the sulfur-substituted Dewar isomers in cells is currently unknown.

Interstrand photocrosslinking with a complementary adenine is also observed in double-stranded DNA oligonucleotides containing 4tThd after UVA radiation *in vivo*.^56^ This cross-linking seems to be independent of sequence, but it is less efficient when 4tThd is flanked by thymine, likely because of the competing intrastrand photocycloaddition reaction with thymine.^56^ As depicted in Fig. 1, DNA–protein crosslinks have also been detected following UVA irradiation of DNA oligonucleotides containing 4tThd^71,72^ or 4tThd-treated bladder cancer cells.^61^ These DNA–protein crosslink lesions are difficult to repair by the DNA repair machinery and have been suggested to be a significant contributor to the cell death by apoptosis upon 4tThd-treatment following UVA irradiation.^54^ Double strand breaks have also been observed in 4tThd-treated cells after UVA irradiation,^56^ and inhibition of DNA replication by other thionated photoproducts has been reported.^73^

The photosensitizing activities of 2,4dtThy, 2,4dtUra, and 2,6dtPu, three of the most promising thiobase derivatives discovered to date (Fig. 2),^47,49^ have been investigated in significantly less detail than those of 4tThd. These derivatives decrease the proliferation of human epidermoid carcinoma cells by up to 63% *in vitro*, only upon activation with a low dose of UVA radiation (5 J cm^−2^).^47^ The increased efficacy of 2,6dtPu compared to 4tThd, 2,4dtThy or 2,4dtUra does not originate from an increase in dark cytotoxicity in the absence of UVA light or from an increase in the production of intracellular ROS. In fact, only a minor increase in the intracellular concentration of ROS is observed for all three thiobases following UVA activation.^47^ While the relative increases in ROS directly correlate with the small singlet-oxygen quantum yields measured in aqueous solution (2,4dtThy > 2,6dtPu > 2,4dtUra), they do not correlate with the observed photodynamic efficacies (2,6dtPu > 2,4dtThy = 2,4dtUra), suggesting these dithiobases can act as effective photosensitizers within oxygen-deficient environments. Transient absorption investigations reveal the underlying photochemical properties of these thiobase derivatives in aqueous solution and rationalize the twofold enhancement of 2,6dtPu as being due to a twofold increase in its triplet-decay lifetime compared with 2,4dtThy and 2,4dtUra.^47^ Collectively, the nominal evidence available suggests that the excited triplet state of these doubly-substituted thiobases can participate in photocycloaddition reactions with adjacent DNA and RNA nucleobases leading to cell death, as has been demonstrated in the case of 4tThd.^43,56,63^

2,4-Dithiouracil has been shown to exhibit a threefold increased rate of photocrosslinking with adenosine monophosphate^74^ compare to the widely used photocrosslinking agent 4-thiouracil. Rational functionalization of these and other thiobase derivatives, aimed at increasing the magnitude of their triplet decay lifetimes and/or their two-photon absorption cross sections (see Section 4),^49^ is expected to further increase the photodynamic efficacy of sulfur-substituted nucleobases and expedite their application in clinical and cosmetic photodynamic therapy. In addition, further investigations are required to delineate the precise photosensitization mechanism by which these doubly-thionated nucleobase derivatives photosensitize damage to cellular DNA.

PDT is generally associated with severe pain and reduced selectivity, even though it is not carcinogenic.^24,25^ ROS are the primary mediators of the pain experience during PDT, contributing to stimulations of sensory neurons that conduct pain to the sensors on the cerebral cortex.^75^ Another associated factor is cell death by necrosis because immune responses such as inflammation can occur.^75^ Thiobase derivatives can act as “*Trojan horses*” of rapidly proliferating tumor cells, further increasing selectivity and cellular DNA/RNA damage, while simultaneously working as effective photosensitizers both in the presence and in the absence of O_2_. Thiobases are also expected to clear quickly from the body because similar DNA derivatives exhibit elimination half-lives of 1–10 hours,^76^ as opposed to the days or weeks of photosensitivity experienced with other photosensitizers.

## 3. Second generation of heavy-atom-free photosensitizers based on thionation of organic molecules

Thiobases exhibit larger molar absorption coefficients at longer wavelengths, larger singlet–triplet spin–orbit coupling constants, and more efficient intersystem crossing to the triplet manifold than the corresponding carbonyl nucleobases.^49,50,77^ However, one of the perceived drawbacks of using thiobases in photodynamic applications is the reduced tissue-penetrating ability of UVA radiation compared to the visible and near infrared radiation presently in use in porphyrin-based PDT.^44^ Hence, it seems sensible that thionation of other biocompatible organic molecules may afford heavy-atom-free photosensitizers for practical PDT applications. In 2019, Nguyen *et al.*^78^ demonstrated that replacing both oxygen atoms in naphthalimide derivatives with sulfur atoms to form thionaphthalimide derivatives result in enhanced intersystem crossing to reactive triplet states that can be used for PDT of HeLa cancer cells under O_2_-deprived (1% O_2_) conditions. It was also shown that the combination of thionation with the introduction of electron-donating groups could be used to increase the potential of developing photosensitizers that generate ROS *via* both type-I and type-II mechanisms at photoactivation wavelengths near the phototherapeutic window. This chemistry was further extended through the incorporation of a dual-functional morpholine group (*i.e.*, electron-donating and lysosome-targeting) into the thionaphthalimide, which was shown to generate ROS in the lysosomes of HeLa cells and to cause cell death upon light irradiation.^79^

**Fig. 5:**
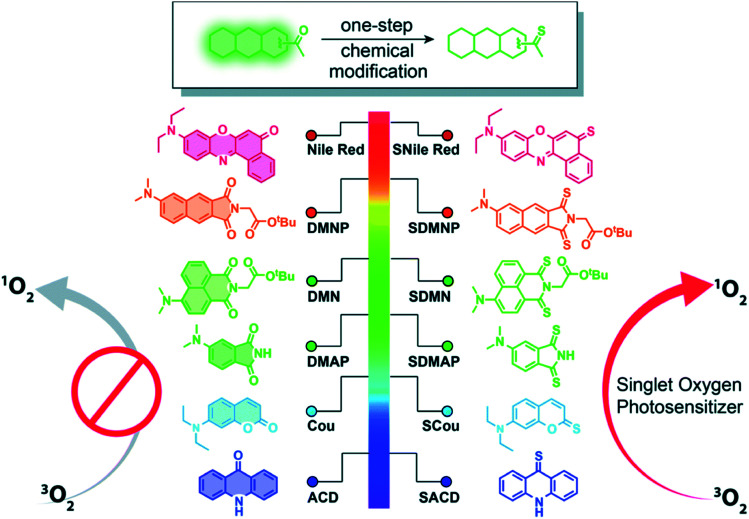
Thionation of a variety of biocompatible organic compounds can dramatically enhance their abilities to generate ROS. Illustrated are structures of the starting organic compounds (left) and thionated photosensitizers (right). Reproduced from ref. 80.

Xiao and co-workers^80,81^ have recently generalized the thionation approach to design heavy-atom-free photosensitizers that can be photoactivated by visible or near-infrared light. Thionation of the carbonyl groups leads to near-unity singlet-oxygen quantum yields and large molar absorption coefficients at wavelengths as long as 760 nm (Fig. 5). The developed thionated photosensitizers do not exhibit cytotoxicity in the dark, while show good photodynamic efficacy against cancer cells and 3D multicellular tumor spheroids.^80^ The authors further conjugated the thionated photosensitizers to the trastuzumab, a monoclonal antibody targeting human epidermal growth factor receptor 2 (HER2), to achieve tumor-specific delivery and demonstrated their tumor-specific therapeutic activity against a HER2-positive cell line. Collectively, these results indicate the promising value of thionated photosensitizers as heavy-atom-free photodynamic agents for PDT applications based on intracellular generation of ROS.^78,79,81^

## 4. Future directions and other prospective applications of thionated photosensitizers

The synergistic cytotoxicity of the thiobases when combined with UVA radiation has been mostly investigated in PDT for skin cancer treatment. However, PDT is established as a potent and less invasive treatment for several other cancers such as lung cancer,^82,83^ esophageal cancer,^84^ gastric cancer,^85^ and cervical cancer.^86^ In order to perform PDT for lung cancer, for example, a bronchoscope is inserted into the airways of the patient. The bronchoscope has a small camera at one of the extremes allowing for the simultaneous visualization of the cancer and selective irradiation of the tumor cells containing the photosensitizer using low-power laser radiation. A similar procedure could be implemented to treat other types of internal cancers that are locally injected with thionated photosensitizers and exposed to laser radiation through an optical fiber. In particular, such a procedure could significantly expand the spectrum of potential uses of thiobases, as well as other biocompatible thionated photosensitizers, in PDT.

Antimicrobial applications of PDT are now growing at a similar or faster rate than anti-cancer applications due to the widespread of multi-drug-resistance pathogenic microbes.^36,87,88^ For example, antimicrobial photoinactivation is used in endodontics for antimicrobial disinfection.^89^ Importantly, antimicrobial photosensitizers are not restricted to have absorption bands in the visible or infrared because most of the infections to be treated are superficial in nature.^36^ Photoinactivation therapy is safe and relatively easy to implement and the drugs used usually rely in the generation of ROS, primarily through type II photosensitization.^36,90^ This non-specificity at the target infection bypasses traditional mechanisms of resistance and minimizes the resistance to the drug itself.^36^ Early investigations with 4tThd have demonstrated a modest antiviral effect following UVA irradiation.^91^ However, to our knowledge, none of the other thionated photosensitizers discussed in this minireview have been studied as prospective antimicrobial or antiviral photodynamics agents.

Thionated DNA and RNA derivatives, have been employed in structural biology applications since their discovery in the mid-1900s due to their photocrosslinking capabilities.^44–46,92^ For example, 4-thiouracil (4tUra) has been widely used in photocrosslinking investigations to gain information about RNA–protein interactions and to uncover *in vivo* RNA structures. Pollum *et al.* showed that 2,4dtUra can outperform 4tUra in its use as a photocrosslinking agent.^32^ 2,4dtUra exhibits a threefold higher photocrosslinking efficiency than 4tUra at 365 nm, thus requiring shorter irradiation times and lower concentrations in photocrosslinking applications. The redshifted absorption spectrum exhibited by this thiobase (Fig. 2) compared to 4tUra, allows excitation at longer wavelengths and thus, enhancing selective excitation and increasing the depth of light penetration into tissue for *in vivo* applications.

Finally, the use of femtosecond laser pulses to excite thiobase derivatives and other thionated photosensitizers with two visible or near-IR photons is a very attractive strategy that needs to be developed. It offers at least two benefits over direct, one-photon absorption: (1) deeper tissue penetration and (2) enhanced spatial selectivity.^40,49^ Longer wavelengths of visible or near infrared radiation penetrate deeper into tissue than shorter wavelengths of light.^36,93^ The need for simultaneous absorption of both photons implies that a very high photon density (of the order of GW cm^−2^) is required and only the photosensitizers located in the laser beam focus can be excited. Increased attention from the organic chemistry community is essential to develop thiobase derivatives and other thionated photosensitizers with enhanced two-photon absorption cross-sections.

## 5. Conclusions

Thionation of nucleic acids and biocompatible organic compounds is a general and effective approach for developing versatile, heavy-atom-free photodynamic therapy agents. These emerging photosensitizers are expected to offer novel therapeutic approaches for the clinical management of cancers, nonmalignant conditions such as psoriasis, and possibly even for antimicrobial photoinactivation applications.

Thionated photosensitizers are here to stay. Besides their promising use in PDT and structural-biology applications, thionated photosensitizers are expected to find a wider-range of applications such as in bioimaging, photovoltaics, and photocatalytic reactions.^94^

## Note added in proof

While the proofreading process of this minireview, Kim, Yoon and co-workers reported a series of sulfur-substituted naphthalimide and coumarin derivatives exhibiting negligible dark toxicity and simultaneous high phototoxicity and singlet oxygen generation toward HeLa cells and HeLa spheroids upon two-photon excitation at 800 nm.^95^ Indeed, diversity-oriented thioxo-1,8-naphthalimides are rapidly emerging as a versatile class of potent DNA photocleavers and promising photosensitizers for cancer therapy.^96^

## Conflicts of interest

There are no conflicts of interest to declare.
